# Sorption Behavior of Hexabromocyclododecanes (HBCDs) on Weihe River Sediment

**DOI:** 10.3390/ijerph17010247

**Published:** 2019-12-30

**Authors:** Xueli Wang, Xiaoyu Yuan, Shengke Yang

**Affiliations:** 1 Key Laboratory of Subsurface Hydrology and Ecological Effects in Arid Region, Ministry of Education, Chang’an University, Xi’an 710054, China; 15129037687@163.com (X.Y.); xueliwang@gmail.com (S.Y.); 2 School of Water and Environment, Chang’an University, Xi’an 710054, China

**Keywords:** affected factors, HBCDs, sediment, sorption, SR-FTIR

## Abstract

The sorption of hexabromocyclododecanes (HBCDs) on sediment affects the fate and transport of HBCDs in rivers. The sorption of HBCDs on sediment from the Weihe River was investigated by performing batch equilibration experiments, and the effects of changing the pH ionic, strength, and humic acid concentration (HA) on sorption were evaluated. The obtained results indicated that fast rather than slow sorption was the dominant process. Nonlinear sorption isotherms were acquired, and the Freundlich (R^2^ 0.94–0.98) and Langmuir (R^2^ 0.95–0.99) models both described the sorption of HBCDs well. The adsorption capacity for α-HBCD, β-HBCD, and γ-HBCD were calculated using the Langmuir model, and were 443.56, 614.29 and 1146.37 mg/kg, respectively. Thermodynamic analysis shows that HBCDs sorption on sediment is a spontaneous exothermic process. HBCDs sorption was affected by the HA concentration and ionic strength. The amounts of HBCDs sorbed to the sediment decreased as the ionic strength increased, and first increased and then decreased as the HA concentration increased. Changes in pH did not clearly affect the sorption of HBCDs. Synchrotron radiation Fourier-transform infrared spectra (SR-FTIR) was used to characterize the adsorption mechanism, and the obtained result indicated that hydrophobic interactions dominated the mechanism involved in HBCDs sorption on sediment.

## 1. Introduction

Hexabromocyclododecanes (HBCDs) is a widely used brominated flame retardant. HBCDs is mainly used in industrial plants to improve the flame resistances of electronic equipment, plastic materials (particularly extruded and expanded polystyrene), and textiles [1,2,3]. Commercial HBCDs contains three diastereoisomers, α-HBCD, β-HBCD, and γ-HBCD. γ-HBCD is the dominant diastereoisomer, and contributes 70–89% of the total HBCD mixture [4,5]. Demand for HBCDs has been increasing since the use of some polybrominated diphenyl ethers was banned in 2004 [6,7]. However, despite the commercial benefits of using HBCD, HBCDs have been classed as persistent organic pollutants under the Stockholm Convention in 2013 [8]. Like many other persistent organic pollutants, HBCDs can be released to the environment during manufacture, use, or disposal and can then accumulate in environmental media, because they are strongly hydrophobic and poorly biodegradable [1,3,9]. HBCDs have been detected in various environmental matrices, including biota, municipal wastewater, sewage sludge, soil, and stream sediment [10,11,12]. HBCDs in the environment can cause toxic effects (e.g., endocrine disruption, liver function disruption, and neurotoxic effects) in biota [13,14,15].

Sediment acts as a sink for organic compounds, and HBCD concentrations in sediment ranging from nanograms to micrograms per gram of sediment have been found. HBCDs can enter sediment after being transported in air or water [9,16]. HBCDs in an aquatic system can sorb to sediment or be degraded or transformed by microbes in sediment [17,18,19,20,21]. Most previous studies of HBCD in the environment have been focused on HBCD concentrations, distributions, toxicities, and degradation [17,22,23]. Sorption on soil or sediment strongly affects organic contaminant transport, degradation, and bioavailability in the environment. Numerous studies of the sorption of nonionizable and ionizable contaminants with relatively high aqueous solubilities to soil have been performed, but few studies of the sorption of nonionizable contaminants with low aqueous solubilities have been performed. HBCDs are hydrophobic organic compounds with low water solubilities (2.1–48.8 μg/L) and relatively high log octanol–water partition coefficients (logKow 5.4–5.8) [2]. Strong hydrophobic interactions between HBCDs and sediment can therefore be expected.

The objective of this study was to investigate the sorption of HBCDs on sediment from Weihe River basin. HBCDs sorption isotherms, mechanisms, and thermodynamics were investigated. The effects of changing the contact time, humic acid (HA) concentration, ionic strength, pH, and temperature on HBCDs sorption were assessed. More importantly, synchrotron radiation Fourier-transform infrared spectra (SR-FTIR) was used to characterize the adsorption mechanism. 

## 2. Materials and Methods

### 2.1. Materials

Standard solutions of native α-HBCD, β-HBCD, and γ-HBCD were purchased from AccuStandard (New Haven, CT, USA). Acetonitrile and methanol (both high-performance liquid chromatography grade) were purchased from Thermo Fisher Scientific (Waltham, MA, USA). Ultrapure water (resistivity 18.2 MΩ cm) was produced using a Milli-Q Integral 15 system (Merck, Darmstadt, Germany). All other chemicals and reagents were of analytical grade.

**Figure 1:**
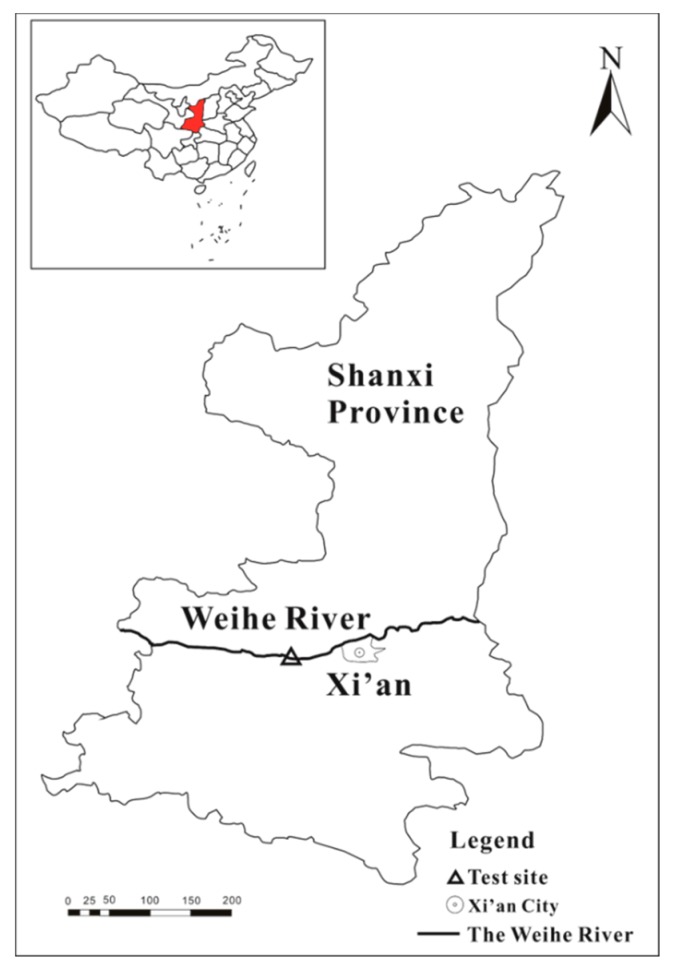
The map of Weihe River and sediment sampling site (∆).

Sediment was collected from the Weihe River Basin, China. The locations of the sampling site are shown in Figure 1. Sediment sample was taken from the sediment surface to 5 cm underground (0–5 cm) using a stainless-steel grab sampler. The sediment was dried in air and then passed through a 0.25 mm sieve. The total organic carbon content was determined using a Vario TOC cube system (Elementar, Germany), and the detected organic carbon content of the sediment was 1.2%. The original sediment sample was stored in a glass vessel until the sorption experiments were performed. 

### 2.2. Sorption Experiments

The batch equilibration method was used to process the sorption experiments. Each test was performed using a 40 mL glass tube with a PTFE (Poly tetra fluoroethylene)-lined screw cap. The background solution (aqueous solution) contained 0.01 M CaCl_2_ and 100 mg/L NaN_3_ in order to maintain constant ionic strength and inhibit microbial activity. A 200 mg sediment sample and 30 mL of the background solution were added to each glass tube in order. HBCDs were added to each tube to give initial HBCD concentrations in the tubes of between 0.01 and 1.0 μg/L [24]. HBCDs are poorly soluble in water, so a high concentration standard in methanol was prepared and then added to the background solution to give the desired concentration. To avoid cosolvent effects, the methanol concentration in the test solution was always <0.1%. The tubes were shaken at 200 rpm for a certain period of time at 25 °C, then centrifuged at 12,000 rpm for 10 min. A 1.0 mL aliquot of the supernatant in each tube was then transferred to a vial for analysis. Control tests (containing the solutes but no sediment) were performed to evaluate the loss of HBCDs in the absence of sediment. Negligible amounts of HBCDs were lost through photochemical decomposition, volatilization, and sorption to the vessel walls.

The kinetics of HBCDs sorption on sediment was investigated using an initial HBCD concentration of 0.5 μg/L at pH 8.0. A 200 mg aliquot of sediment was added to each of a series of tubes containing 30 mL of the HBCDs solution. The tubes were shaken vigorously in an incubator at 298 K. An aliquot of the solution in a tube was withdrawn at each of a series of specified times during a test. The aliquot was centrifuged and analyzed, and the amounts of HBCDs sorbed to the sediment, $q_{t}$(mg/kg), were calculated using Equation (1):

(1) $$q_{t}=\frac{\left(C_{0}-C_{t}\right)V}{m}$$

where *C*_0_ is the initial HBCDs concentration (mg/L), *C_t_* is the HBCDs concentration (mg/L) at time *t*, *V* is the volume of HBCDs solution used (L), and m is the sediment mass (kg).

The effect of changing the temperature on HBCDs sorption on sediment was investigated by performing tests at temperatures between 298 and 318 K. The effect of changing the pH on HBCDs sorption on sediment was investigated by performing tests using solutions between pH 4.0 and 10.0. A series of tubes each containing sediment and a HBCDs solution at the desired concentrations and pH were shaken until equilibrium was judged to have been reached. The tubes were then centrifuged, then the pH of the solution in each tube and the HBCDs concentration in the supernatant were determined.

The effect of changing the ionic strength of the solution on HBCDs sorption on sediment was investigated using sodium bicarbonate to give ionic strengths between 0.04 and 0.36 mol/L. Solutions at the desired initial ionic strengths and containing HBCDs at the desired concentrations were prepared. The effect of changing the HA concentration on HBCDs sorption on sediment was investigated in a similar way but using HA concentrations between 1.0 and 30.0 mg/L.

### 2.3. Analytical Methods

The α-HBCD, β-HBCD, and γ-HBCD concentrations in each sample were determined using a high-performance liquid chromatography coupled to a triple quadrupole mass spectrometer (Agilent 6470 TSQ). The mass spectrometer was operated in electrospray negative ionization mode, and the method used was based on previously published methods [25,26]. The detailed analytical methods can be found in the support information (Figure S1–S5).

## 3. Results and Discussion

### 3.1. Sorption Kinetics

**Figure 2:**
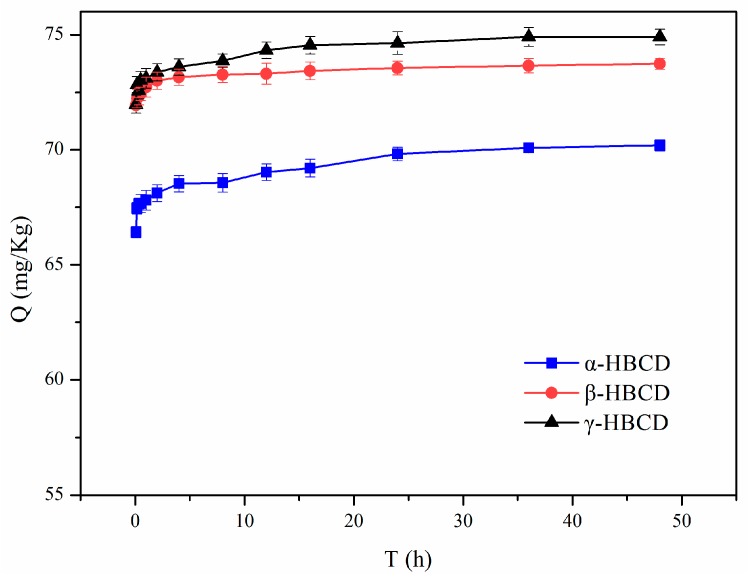
Sorption equilibrium time of hexabromocyclododecanes (HBCDs) in the sediment.

The kinetics of the HBCDs sorption on sediment is shown in Figure 2. HBCDs sorption on sediment had a fast step (<30 min) and then a slow step. Extremely rapid sorption of HBCDs occurred during the first 10 min. The amounts of α-HBCD, β-HBCD, and γ-HBCD sorbed reached 92.9%, 95.8%, and 96.0%, respectively, of the maximum sorption capacities in the fast sorption period. At 2 h, the amounts of α-HBCD, β-HBCD, and γ-HBCD sorbed had reached 97.0%, 98.9%, and 97.9%, respectively, of the maximum sorption capacities. Once the fast sorption step was complete, desorption would have started to become important, and the amounts of the HBCDs that sorbed to the sediment changed more slowly. Sorption equilibrium appeared to have been reached at ~24 h. These results indicated that fast sorption rather than slow sorption was the dominant mechanism. Fast HBCDs sorption could probably be attributed to sorption of HBCDs to mineral surfaces or the partitioning of HBCDs into amorphous organic matter [24,27,28], and slow sorption could be attributed to gradual diffusion of HBCDs into organic matter and sediment micropores [29]. These results suggest that the dissolution and partitioning of HBCDs into organic matter in the sediment may play important roles in the sorption of HBCDs. We concluded that equilibrium was achieved in 24 h, so subsequent tests were performed using a 24 h equilibration time.

### 3.2. Sorption Isotherms

The HBCD–sediment equilibrium data were modeled using Langmuir and Freundlich isotherm models.

The Langmuir isotherm model can be expressed as:

(2) $$\frac{C_{e}}{q_{e}}=\frac{C_{e}}{q_{max}}+\frac{1}{q_{max}K_{L}}$$

where *q_e_* (mg/kg) is the amount of HBCD sorbed at equilibrium, *C_e_* (μg/L) is the HBCD concentration at equilibrium, *q_max_*(mg/kg) is the maximum adsorption capacity calculated using the Langmuir model, and *K_L_* (L/μg) is the Langmuir constant. The dimensionless constant separation factor for the equilibrium parameter (*R_L_*) used in the model was determined using the equation:

(3) $$R_{L}=\frac{1}{1+K_{L}C_{0}}$$

where *C*_0_ (μg/L) is the initial HBCD concentration. The *R_L_* value was used to indicate the type of isotherm, *R_L_* = 0 indicating irreversible sorption, 0 < *R_L_* < 1 indicating favorable sorption, *R_L_* = 1 indicating linear sorption kinetics, and *R_L_* > 1 indicating unfavorable sorption.

The Freundlich isotherm model can be expressed as:

(4) $$q_{e}=K_{f}C_{e}^{n}$$

where *K_f_* ((mg/kg)/(μg/L)n) is the Freundlich constant and *n* (dimensionless) is the Freundlich intensity parameter. The validity of each isotherm model was assessed from the correlation coefficient (R^2^) obtained.

**Figure 3:**
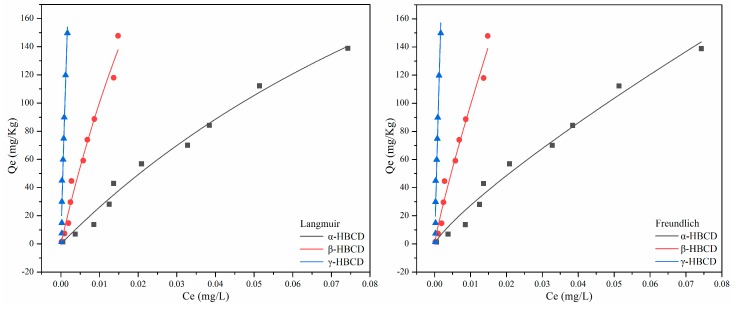
Sorption isotherms of HBCDs in the sediment.

The data from the sorption equilibrium tests for the three HBCD isomers are shown in Figure 3. It can be seen that when the initial HBCD concentration increased from 10 to 1000 μg/L, the amounts of α-HBCD, β-HBCD, and γ-HBCD sorbed per unit of sediment increased from 1.42 to 140.41, 1.47 to 146.76, and 1.46 to 149.69 mg/kg, respectively.

The isotherm models fitted to the data and the calculated parameters are shown in Table 1. The Langmuir and Freundlich models both performed well and gave high R^2^ values, but the data were fitted slightly better by the Langmuir isotherm model than the Freundlich isotherm model, and the residual sum of squares was lower for the Langmuir model than the Freundlich model. The maximum adsorption capacities for α-HBCD, β-HBCD, and γ-HBCD at 298 K, calculated using the Langmuir model, were 443.56, 614.29, and 1146.37 mg/kg, respectively. The *R_L_* values for α-HBCD, β-HBCD, and γ-HBCD, calculated using Equation (2), were 0.243, 0.093, and 0.021, respectively. These results indicated that adsorption of HBCD to sediment was favorable. In the Freundlich model, *K_f_* is the adsorption capacity parameter. The adsorption capacity of an adsorbent will generally increase as *K_f_* increases. The α-HBCD, β-HBCD, and γ-HBCD K*_f_* values were 1233.43, 5424.61, and 71,870.33 (mg/kg)/(μg/L)n, respectively. The sediment had a higher adsorption capacity for γ-HBCD than the other isomers, the second highest adsorption capacity was for β-HBCD, and the lowest adsorption capacity was for α-HBCD. This agreed with the Langmuir model and experimental data.

### 3.3. Sorption Thermodynamics

The thermodynamics of the sorption of HBCDs on sediment were investigated to determine the energy changes occurring during sorption. The studies were carried out at 298 K, 308 K and 318 K, respectively. The standard enthalpy change (ΔH_0_, in kJ/mol), standard entropy change (ΔS_0_, in J/(mol K)), and standard Gibbs free energy change (ΔG_0_, in kJ/mol) were calculated using Equations (5) and (6), and the results are shown in Table 2.

(5) $$\Delta G=-RT\ln K_{d}$$

(6) ΔG=ΔH−TΔS

In Equations (5) and (6), *K_d_* (L/g) is the distribution coefficient and R (8.314 J/(mol K)) is the universal gas constant. The sorption equilibrium constants can be achieved by the following method: as the concentration of HBCD decreases and approaches 0, values of *K_d_* are calculated by plotting a straight line of (*q_e_/C_e_*) versus q_e_ based on extrapolating q_e_ to zero. The value of the intercept is that of *K_d_*.

The calculated parameters are shown in Table 2. The standard Gibbs free energy was negative for all the HBCD isomers at the test temperatures, indicating that α-HBCD, β-HBCD, and γ-HBCD sorption on sediment was a thermodynamically feasible and spontaneous process. The standard Gibbs free energy changes for α-HBCD, β-HBCD, and γ-HBCD were similar, and increased as the temperature was increased from 298 to 318 K, indicating that the temperature affected HBCD sorption only slightly. The standard enthalpy changes were negative, indicating that the HBCD sorption on sediment was exothermic.

### 3.4. Effect of Temperature on HBCD Sorption

The temperature will, generally, strongly affect sorption. Tests were performed to investigate α-HBCD, β-HBCD, and γ-HBCD sorption on sediment at temperatures between 298 and 318 K. As shown in Figures S1–S3, the amounts of α-HBCD, β-HBCD, and γ-HBCD sorbed to sediment decreased as the temperature increased from 298 to 318 K. This implied that the HBCD sorption on the sediment was exothermic.

### 3.5. Effect of pH on HBCD Sorption

**Figure 4:**
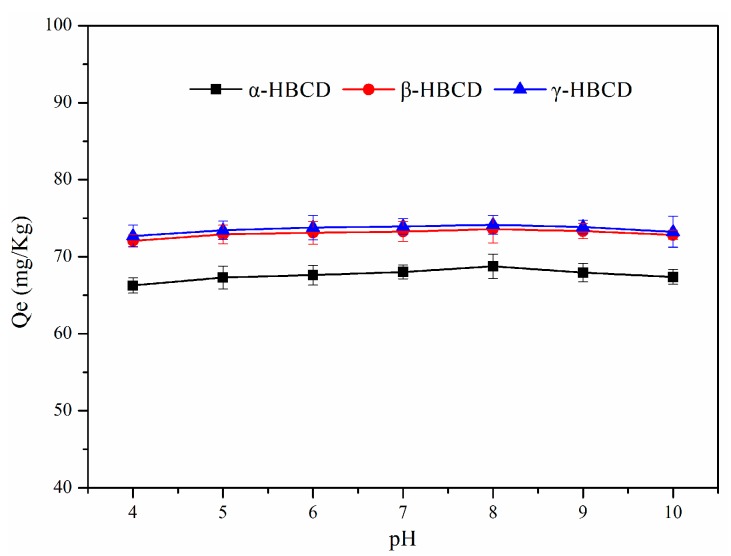
Effects of pH on HBCDs sorption in the sediment.

Tests were performed to investigate the sorption of α-HBCD, β-HBCD, and γ-HBCD on sediment at pH values between 4 and 10. The results are shown in Figure 4. Increasing the pH only minorly affected α-HBCD and β-HBCD sorption on sediment and slightly affected γ-HBCD sorption on sediment. This is because HBCDs are strongly hydrophobic, meaning hydrophobic mechanisms will dominate the adsorption process. Changing the pH does not strongly affect such hydrophobic mechanisms.

### 3.6. Effect of the HA Concentration on HBCD Sorption

**Figure 5:**
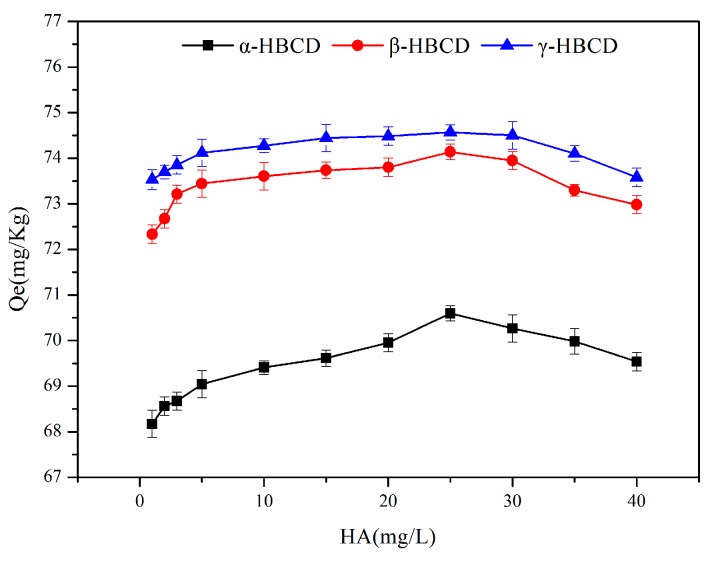
Effects of humic acid (HA) on HBCDs sorption in the sediment.

Dissolved organic matter in river water is mainly humus, carbohydrates, and proteins. These materials are also generally found in sediment and soil. Dissolved organic matter contains extremely active components in terms of pollutant behavior, and strongly affects organic pollutant migration, transformation, and final destination in rivers. Tests were performed using HA to represent dissolved organic matter. Tests were performed to investigate α-HBCD, β-HBCD, and γ-HBCD sorption on sediment at different HA concentrations. As shown in Figure 5, the amounts of all three HBCD isomers sorbed to sediment first increased and then decreased as the HA concentration increased. This may have been because HBCD sorption on sediment was affected by the total organic carbon content of the sediment. Adding HA to the solution would have indirectly increased the total organic carbon content of the sediment and therefore increased the amounts of HBCDs that sorbed to the sediment. At HA concentrations >25 mg/L, the HA would have occupied sorption sites on the sediment that would otherwise have been available to HBCDs, and therefore inhibited HBCD sorption.

### 3.7. Effect of the Ionic Strength on HBCD Sorption

**Figure 6:**
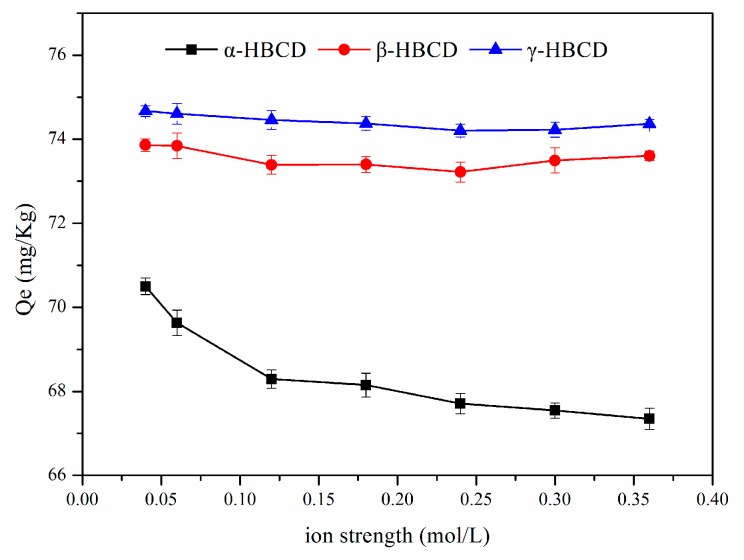
Effects of ion strength on HBCDs sorption in the sediment.

It has previously been found that the amount of a species sorbed can decrease, increase, or remain unchanged as the ionic strength increases, and that changing the ionic strength can change the adsorption kinetics. Tests were performed using different NaHCO_3_ concentrations to investigate the relationships between the ionic strength and α-HBCD, β-HBCD, and γ-HBCD sorption on sediment. The results are shown in Figure 6. Increasing the ionic strength decreased the amount of α-HBCD sorbed to sediment but only slightly affected the amounts of β-HBCD and γ-HBCD sorbed to sediment. This would have been because HBCDs are strongly hydrophobic, and hydrophobic mechanisms would have dominated HBCD sorption on sediment. However, repulsive electrostatic interactions between HBCD molecules and the negatively charged sediment surface would have caused the slight decrease in the amounts of HBCDs sorbed as the ionic strength increased.

### 3.8. Sorption Mechanism

The kinetics experiments indicated that the sorption processes were fast. Fast sorption is usually attributed to physical adsorption. The adsorption mechanism was investigated by analyzing the sediment with and without HBCDs sorbed to it by Fourier-transform infrared (FTIR) and synchrotron radiation (SR) FTIR spectroscopy. Pure HBCDs were also analyzed by FTIR and SR-FTIR spectroscopy. The FTIR spectra of sediment before and after HBCD sorption are shown in Figure S4a,b. The FTIR spectra of pure HBCDs are shown in Figure S4c. The same peaks were found between 800 and 4000 cm^−1^ for sediment with and without HBCDs sorbed.

**Figure 7:**
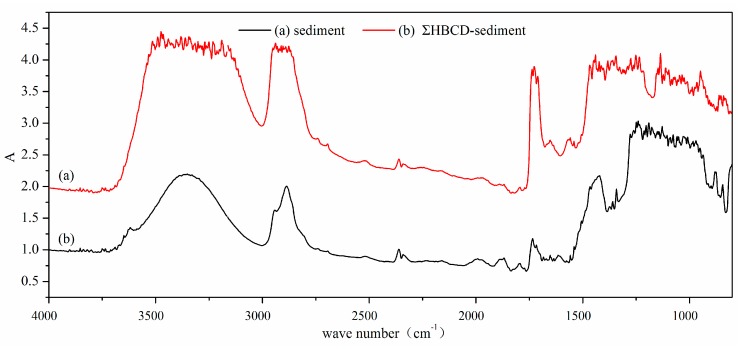
SR-FTIR spectra of (**a**) sediment, (**b**) ∑HBCD-sediment.

The SR-FTIR spectra for sediment before and after HBCD sorption are shown in Figure 7a,b. The SR-FTIR spectrum for sediment with small amounts of HBCDs sorbed were markedly different from the spectrum for sediment that had not been exposed to HBCDs. Marked differences were found in the spectra for sediment with and without HBCDs sorbed at 1000–1300 cm^−1^ (C–C bending) and 2800–3000 cm^−1^ (–CH2 stretching). Less absorbance was found at 2800–3000 cm^−1^ for sediment with HBCDs sorbed than for sediment that had not been exposed to HBCDs, and a peak at 1000–1300 cm^−1^ was shifted to the wavelet number for sediment with HBCDs sorbed. These spectral changes may have been caused by the hydrophobicities of HBCDs causing strong adsorption reactions between the HBCDs and sediment.

## 4. Conclusions

The sorption of HBCDs to sediment was investigated. Fast sorption was found to be important to HBCD sorption on sediment. Nonlinear sorption isotherms were found, and the Langmuir and Freundlich models both described HBCD sorption on sediment well. The maximum sorption capacities for α-HBCD, β-HBCD, and γ-HBCD at 298 K were 443.56, 614.29, and 1146.37 mg/kg, respectively. The α-HBCD, β-HBCD, and γ-HBCD K_F_ values were 1233.43, 5424.61, and 71,870.33 (mg/kg)/(μg/L)n, respectively. HBCD sorption was affected by the HA concentration and the ionic strength. The amounts of HBCDs sorbed decreased as the ionic strength increased and first increased and then decreased as the HA concentration increased. Changing the pH did not significantly affect HBCD sorption. The SR-FTIR spectra indicated that hydrophobic interactions are the main mechanisms through which HBCDs sorb to sediment. The obtained results will improve our understanding of the behaviors of HBCDs in sediment and will benefit assessments of the risks posed by HBCDs in the environment and models of the fates of HBCDs in the environment. At the same time, these results will also provide certain theoretical support for the treatment of HBCD pollution in rivers.

## Figures and Tables

**Table 1:** Isotherm parameters for HBCDs sorption on sediment.

		Langmuir Model Parameter				Freundlich Model Parameter			
	Q_m_ (mg/Kg)	K_L_	R^2^	RSS/dof	R_L_	K_F_	n	R^2^	RSS/dof
α-HBCD	443.56	6.23	0.9867	32.05	0.243	1233.43	0.83	0.9805	41.53
β-HBCD	614.29	19.54	0.9766	62.72	0.093	5424.61	0.87	0.9737	62.73
γ-HBCD	1146.37	91.41	0.9486	141.89	0.021	71,870.33	0.96	0.9388	150.08

**Table 2:** Thermodynamic parameters of HBCDs sorption on sediment.

	T(K)	K	ΔG (kJ·mol^−1^)	ΔH (kJ·mol^−1^)	ΔS (kJ·mol^−1^)
α-HBCD	298	3127.58	−19.94	−49.08	−0.0975
	308	2341.07	−19.22		
	318	1426.58	−17.99		
β-HBCD	298	25286.73	−25.12	−64.87	−0.1335
	308	14274.37	−23.70		
	318	8598.41	−22.45		
γ-HBCD	298	293639.20	−31.19	−75.40	−0.1480
	308	183989.34	−30.03		
	318	88950.41	−28.23		

## Supplementary Materials

The following are available online at https://www.mdpi.com/1660-4601/17/1/247/s1. The detailed analytical methods, Figure S1: Sorption isotherms of α-HBCD in the sediment at different temperatures, Figure S2: Sorption isotherms of β-HBCD in the sediment at different temperatures, Figure S3: Sorption isotherms of γ-HBCD in the sediment at different temperatures, Figure S4: Effects of pH on HBCDs sorption in the sediment, Figure S5: FTIR spectra of (a) ∑HBCD, (b) sediment, (c) ∑HBCD-sediment.

## References

[1] Alaee M., Arias P., Sjödin A., Bergman Å. (2003). An overview of commercially used brominated flame retardants, their applications, their use patterns in different countries/regions and possible modes of release. Environ. Int..

[2] Marvin C.H., Tomy G.T., Armitage J.M., Arnot J.A., McCarty L., Covaci A., Palace V. (2011). Hexabromocyclododecane: Current understanding of chemistry, environmental fate and toxicology and implications for global management. Environ. Sci. Technol..

[3] Rani M., Shim W.J., Han G.M., Jang M., Song Y.K., Hong S.H. (2014). Hexabromocyclododecane in polystyrene based consumer products: An evidence of unregulated use. Chemosphere.

[4] Covaci A., Gerecke A.C., Law R.J., Voorspoels S., Kohler M., Heeb N.V., Leslie H., Allchin C.R., Boer J.D. (2006). Hexabromocyclododecanes (HBCDs) in the Environment and Humans: A Review. Environ. Sci. Technol..

[5] Law R.J., Kohler M., Heeb N.V., Gerecke A.C., Schmid P., Voorspoels S., Covaci A., Becher G., Janák K., Thomsen C. (2005). Hexabromocyclododecane Challenges Scientists and Regulators. Environ. Sci. Technol..

[6] Wang W., Choo G., Cho H.S., Park K., Shin Y.J., Oh J.E. (2018). The occurrence and distribution of hexabromocyclododecanes in freshwater systems, focusing on tissue-specific bioaccumulation in crucian carp. Sci. Total Environ..

[7] Guerra P., Cal A.D.L., Marsh G., Eljarrat E., Barceló D. (2009). Transfer of hexabromocyclododecane from industrial effluents to sediments and biota: Case study in Cinca river (Spain). J. Hydrol..

[8] United Nations Environment Programme (2013). United Nations Environment Annual Repor.

[9] Zhang Y., Lu Y., Wang P., Li Q., Zhang M., Johnson A.C. (2018). Transport of Hexabromocyclododecane (HBCD) into the soil, water and sediment from a large producer in China. Sci. Total. Environ..

[10] Law R.J., Covaci A., Harrad S., Herzke D., Abdallah M.A.E., Fernie K., Toms L.-M.L., Takigami H. (2014). Levels and trends of PBDEs and HBCDs in the global environment: Status at the end of 2012. Environ. Int..

[11] Drage D., Mueller J.F., Birch G., Eaglesham G., Hearn L.K., Harrad S. (2015). Historical trends of PBDEs and HBCDs in sediment cores from Sydney estuary, Australia. Sci. Total Environ..

[12] Cao X., Lu Y., Zhang Y., Khan K., Wang C., Baninla Y. (2018). An overview of hexabromocyclododecane (HBCDs) in environmental media with focus on their potential risk and management in China. Environ. Pollut..

[13] Abdallah M.A., Uchea C., Chipman J.K., Harrad S. (2014). Enantioselective biotransformation of hexabromocyclododecane by in vitro rat and trout hepatic sub-cellular fractions. Environ. Sci. Technol..

[14] Kim U.J., Oh J.E. (2014). Tetrabromobisphenol A and hexabromocyclododecane flame retardants in infant-mother paired serum samples, and their relationships with thyroid hormones and environmental factors. Environ. Pollut..

[15] Miller I., Serchi T., Cambier S., Diepenbroek C., Renaut J., Van der Berg J.H., Kwadijk C., Gutleb A.C., Rijntjes E., Murk A.J. (2016). Hexabromocyclododecane (HBCD) induced changes in the liver proteome of eu- and hypothyroid female rats. Toxicol. Lett..

[16] Wu M.H., Zhu J.Y., Tang L., Liu N., Peng B.Q., Sun R., Xu G. (2014). Hexabromocyclododecanes in surface sediments from Shanghai, China: Spatial distribution, seasonal variation and diastereoisomer-specific profiles. Chemosphere.

[17] Oh J.K., Kotani K., Managaki S., Masunaga S. (2014). Levels and distribution of hexabromocyclododecane and its lower brominated derivative in Japanese riverine environment. Chemosphere.

[18] Xu J., Zhang Y., Guo C., He Y., Li L., Meng W. (2013). Levels and distribution of tetrabromobisphenol A and hexabromocyclododecane in Taihu Lake, China. Environ. Toxicol. Chem..

[19] Li B., Yao T., Sun H., Zhang Y., Yang J. (2016). Diastereomer- and enantiomer-specific accumulation, depuration, bioisomerization, and metabolism of hexabromocyclododecanes (HBCDs) in two ecologically different species of earthworms. Sci. Total Environ..

[20] Zhu H., Sun H., Yao Y., Wang F., Zhang Y., Liu X. (2017). Fate and adverse effects of hexabromocyclododecane diastereoisomers (HBCDDs) in a soil-ryegrass pot system. Chemosphere.

[21] Li B., Zhu H., Sun H., Xu J. (2017). Effects of the amendment of biochars and carbon nanotubes on the bioavailability of hexabromocyclododecanes (HBCDs) in soil to ecologically different species of earthworms. Environ. Pollut..

[22] Jeong G.H., Hwang N.R., Hwang E.H., Lee B.C., Yoon J. (2014). Hexabromocyclododecanes in crucian carp and sediment from the major rivers in Korea. Sci. Total Environ..

[23] Al-Odaini N.A., Shim W.J., Han G.M., Jang M., Hong S.H. (2015). Enrichment of hexabromocyclododecanes in coastal sediments near aquaculture areas and a wastewater treatment plant in a semi-enclosed bay in South Korea. Sci. Total Environ..

[24] Sun Z., Yu Y., Mao L., Feng Z., Yu H. (2008). Sorption behavior of tetrabromobisphenol A in two soils with different characteristics. J. Hazard. Mater..

[25] Li F., Jin J., Tan D., Wang L., Geng N., Cao R., Gao Y., Chen J. (2016). Hexabromocyclododecane and tetrabromobisphenol A in sediments and paddy soils from Liaohe River Basin, China: Levels, distribution and mass inventory. J. Environ. Sci..

[26] Zhao Y., Li Q., Miao X., Huang X., Li B., Su G., Zheng M. (2017). Determination of hexabromocyclododecanes in sediments from the Haihe River in China by an optimized HPLC-MS-MS method. J. Environ. Sci..

[27] Huang W., Schlautman M., Weber W.J. (1996). A Distributed Reactivity Model for Sorption by Soils and Sediments. 5. The Influence of Near-Surface Characteristics in Mineral Domains. Environ. Sci. Technol..

[28] Weber W.J., Huang W. (1996). A Distributed Reactivity Model for Sorption by Soils and Sediments. 4. Intraparticle Heterogeneity and Phase-Distribution Relationships under Nonequilibrium Conditions. Environ. Sci. Technol..

[29] Pignatello J., Xing B. (1996). Mechanisms of Slow Sorption of Organic Chemicals to Natural Particles. Environ. Sci. Technol..

