# Supplementary material for: Sorption Behavior of Hexabromocyclododecanes (HBCDs) on Weihe River Sediment
Source: Int J Environ Res Public Health. 2019 Dec 30;17(1):247. doi: 10.3390/ijerph17010247 (PMC6981516; doi:10.3390/ijerph17010247)
Supplement: Supplementary file 1 [file ijerph-17-00247-s001.pdf]

Sorption behavior of hexabromocyclododecanes (HBCDs) on Weihe River sediment

Xueli Wang[1,2]*, Xiaoyu Yuan[2], Shengke Yang[1,2]

1. Key Laboratory of Subsurface Hydrology and Ecological Effects in Arid Region, Ministry of Education, Chang'an University, Xi'an 710054, China.
2. School of Water and Environment, Chang'an University, Xi'an 710054, China.
3. *Correspondence: wxl0120@126.com(X.W.);Tel.: +86-189-9130-0706

Analytical methods

The α-HBCD, β-HBCD, and γ-HBCD concentrations in each sample were determined using a high-performance liquid chromatography coupled to a triple quadrupole mass spectrometer (Agilent 6470 TSQ). The HBCDs stereoisomers were separated using a C18 column (2.1 mm i.d., 150 mm long, 3.0 μm particle size; Agilent, USA). During an analytical run, the column temperature was kept at 40 °C. The injection volume was 5.0 μL. Three mobile phases were used (A) acetonitrile, (B) methanol, and (C) water, and the flow rate was 0.3 mL/min. The mobile phase gradient program started at an A/B/C ratio of 55/20/25 (v/v/v), then changed in a linear fashion to an A/B/C ratio of 70/20/10 (v/v/v) over a period of 12.0 min, then changed to an A/B/C ratio of 100/0/00 (v/v/v) over 0.2 min. This A/B/C ratio was maintained for 8 min, then the A/B/C ratio changed to 55/20/25 (v/v/v), which was maintained for 9 min.

The mass spectrometer was operated in electrospray negative ionization mode, and the method used was based on previously published methods [24, 25]. The triple quadrupole mass spectrometer was operated in selected reaction monitoring mode. The capillary temperature and capillary spray voltage were 230 °C and 3.0 kV, respectively. The sheath gas and auxiliary gas were nitrogen, and the pressures were 28 psi and 5 psi, respectively. The tube lens offset was 80. The $[M-H]^{-} \rightarrow Br^{-}$ transitions m/z 640.2 → 81.0/642.2 → 81.0 were monitored.

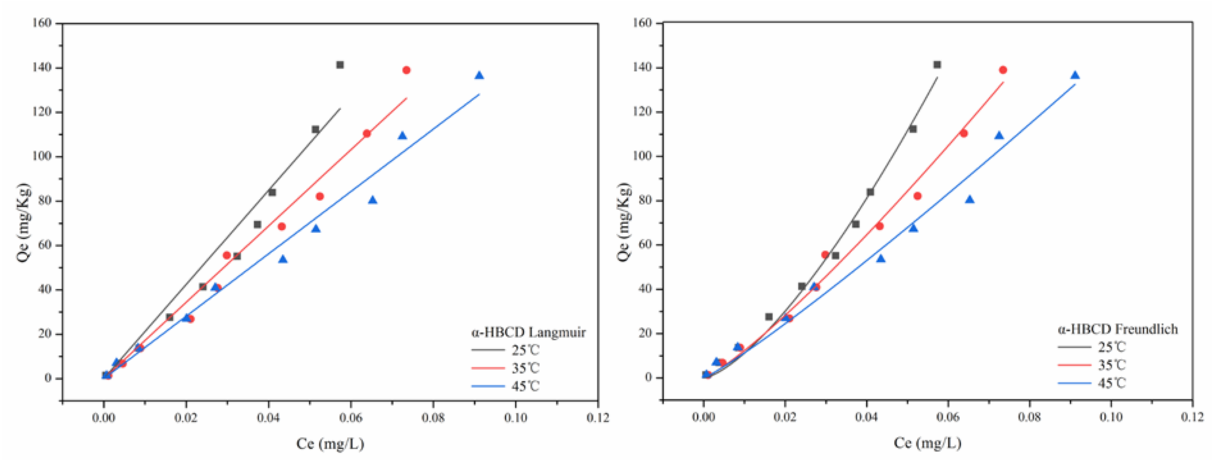

Figure S1. Sorption isotherms of α-HBCD in the sediment at different temperatures.

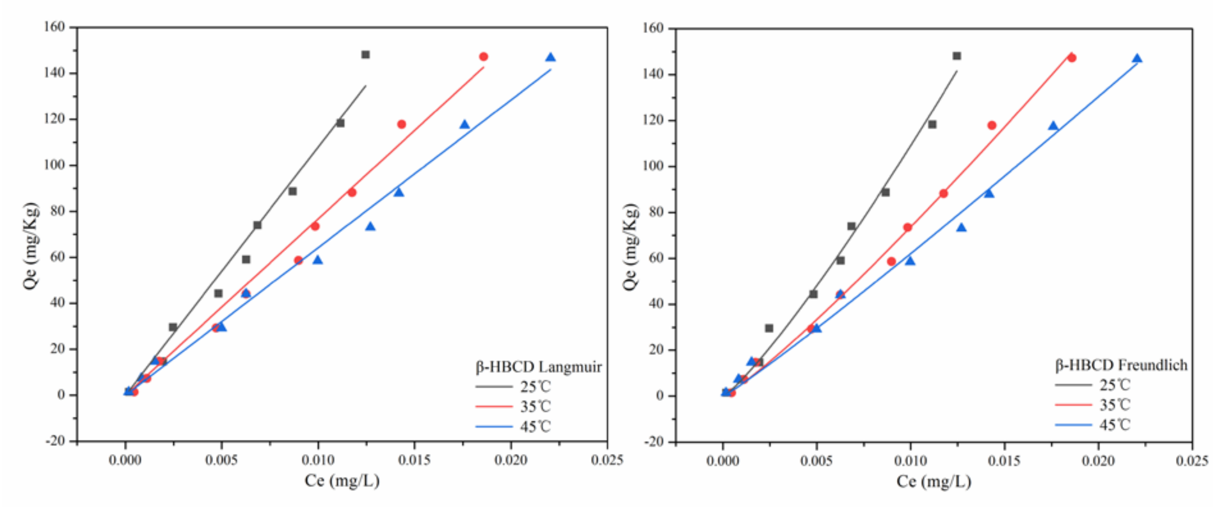

Figure S2. Sorption isotherms of β-HBCD in the sediment at different temperatures.

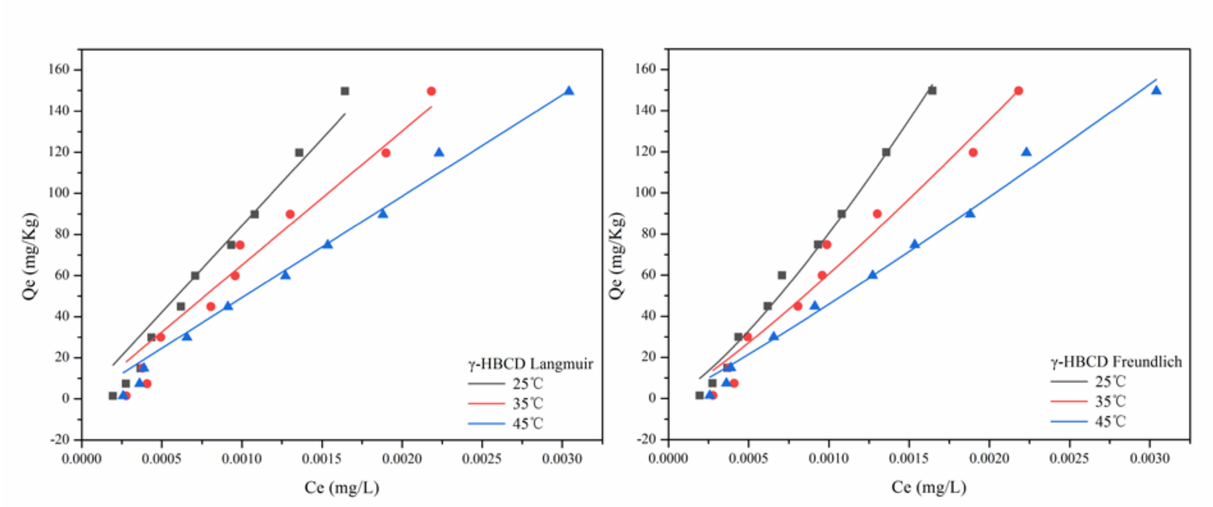

Figure S3. Sorption isotherms of γ-HBCD in the sediment at different temperatures.

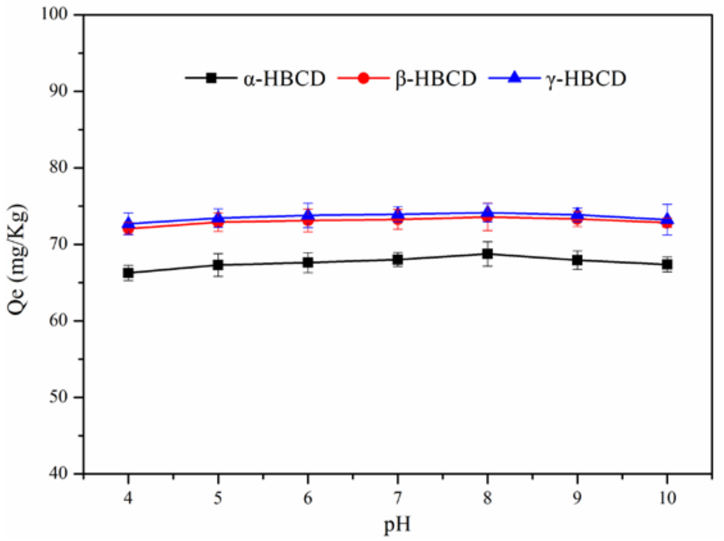

Figure S4. Effects of pH on HBCDs sorption in the sediment.

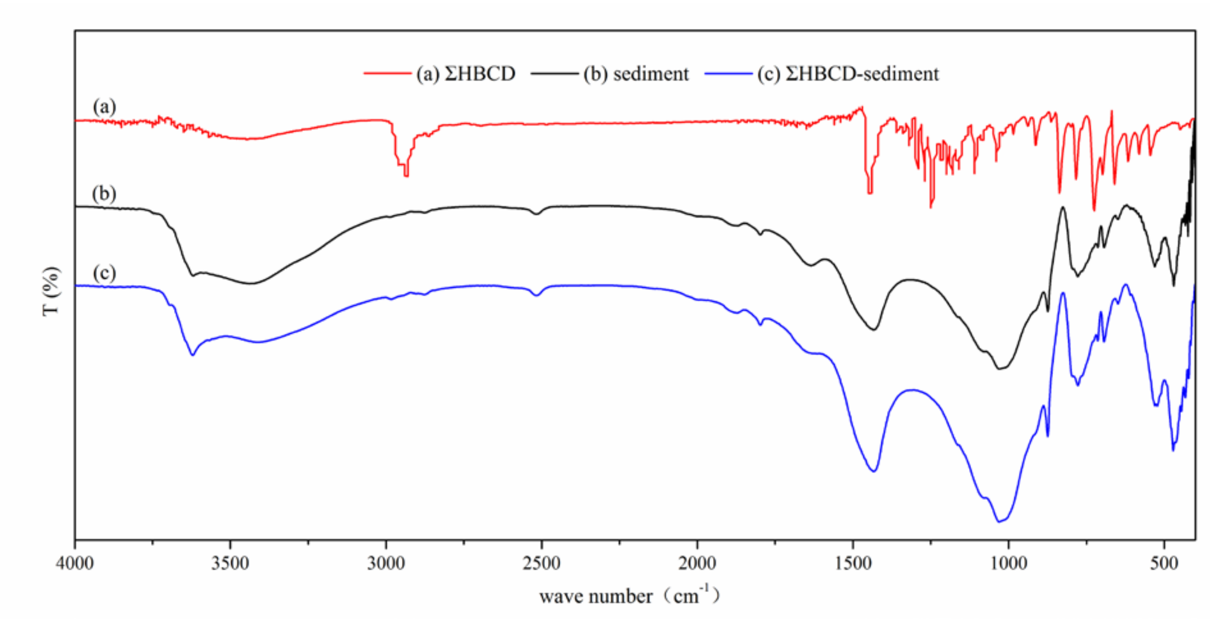

Figure S5. FTIR spectra of (a)∑HBCD, (b)sediment, (c)∑HBCD-sediment.
